# A systematic review and meta-analysis of the globally reported International Classification of Diseases to Perinatal Mortality (ICD-PM)

**DOI:** 10.3389/fmed.2024.1434380

**Published:** 2024-09-23

**Authors:** Henok Kumsa, Esuyawkal Mislu, Nigus Bililign Yimer

**Affiliations:** School of Midwifery, College of Health Sciences, Woldia University, Woldia, Ethiopia

**Keywords:** ICD-PM, stillbirth, neonatal death, perinatal mortality, meta-analysis

## Abstract

**Introduction:**

Accurate recording and identification of perinatal mortality causes are crucial to reducing the global burden of perinatal mortality through targeted interventions. However, existing studies on the International Classifications of Diseases to Perinatal Mortality (ICD-PM) are limited by inconsistent results and variations by gestational age. Thus, this review aims to synthesize and document updated data on the causes of death using the ICD-PM classification.

**Methods:**

Electronic databases such as the PubMed via MEDLINE, SCOPUS, Web of Sciences, EMBASE, Cochrane Library, and PROSPERO were searched to retrieve studies published from 2016 to February 2024. The Newcastle–Ottawa Scale (NOS) was used to assess the quality of the included studies, and heterogeneity between the studies was assessed using I^2^ statistics. ICD-PM coded reported data were extracted to Microsoft Excel, and aggregate data of frequencies and percentages were reported.

**Results:**

Out of the 23 included studies, 48,596 perinatal mortalities were reported, and approximately 96% (46,816 deaths) were classified according to the ICD-PM. The pooled rate of stillbirths in high-income countries was 23/1,000 births; in low-income countries, it was found to be approximately twice as in high-income countries. Regarding the category of deaths, 25,563 (54.6%) deaths were recorded in the antepartum period, and more than half, 14,887 (58.2%), were classified under unspecified causes (A6). Moreover, 6,148 (13.7%) and 14,835 (31.7%) deaths were coded with intrapartum and neonatal period causes, respectively. The leading causes of perinatal mortality during the intrapartum were acute intrapartum events (I3) 3,712 (57.8%). Furthermore, neonatal death was caused by low birth weight and prematurity (N9) 4,091 (27.6%), congenital malformations, and chromosomal abnormalities (N1) 2,512(16.9%).

**Conclusion:**

Congenital malformations, and chromosomal abnormalities contribute to 1 in every 10 perinatal deaths and 1 in every 4 neonatal deaths. Other specified antepartum disorders are responsible for over half of antepartum deaths, while acute intrapartum events are the leading cause of intrapartum deaths, with a significant proportion remaining unexplained. Maternal complications related to the placenta, membranes, cord, labor, and delivery play a significant role in antepartum and intrapartum deaths. Targeted interventions and improved monitoring of high-risk pregnancies are crucial to reducing perinatal mortality rates. Further investigation is needed to enhance understanding and address unexplained perinatal deaths.

**Systematic review registration:**

[https://clinicaltrials.gov/], identifier [CRD4202452549].

## 1 Introduction

Perinatal mortality is defined as the loss of fetuses at or beyond 20 weeks, deaths during labor and delivery, as well as early neonatal deaths (1). Globally, the stillbirth rate is estimated to be 13.9/1,000 births, resulting in approximately 2.6 million stillbirths annually (2, 3). Singapore and Finland have the lowest stillbirth rates, with only 2/1,000 births. In sub-Saharan Africa, the stillbirth rate is estimated at 21/1,000 births (3, 4). Notably, low-income countries account for approximately 98% of the reported stillbirths (5). Perinatal deaths have been insufficiently documented, and large variations exist across regions (4, 5).

Perinatal mortality can have far-reaching social, psychological, economic, and medical consequences (6). Consequently, perinatal death imposes a substantial burden on societies as it can have unforeseen negative consequences for families (6, 7). Additionally, the perinatal mortality rate is an important indicator of a country’s development. Therefore, analyzing perinatal deaths is valuable for clinicians and policymakers, helping identify areas for improvement and shaping effective policies. However, many countries, specifically low- and middle-income countries, have no national registration for perinatal mortality (8, 9).

A narrative review showed that over 81 different classification systems were used to categorize perinatal deaths between 2009 and 2014 (10), such as ReCoDe, INCODE, and TULIP (11–13). These systems often require additional evidence, such as histological findings or post-mortem examinations, to support certain diagnoses (13). Furthermore, some of these classification systems were developed using specific, computerized systems and programs to record patient information, and the application of these systems may also require the use of similar technological tools (14, 15).

In 2016, the World Health Organization (WHO) introduced the International Classification of Diseases to Perinatal Mortality (ICD-PM), a standardized system designed to uniformly identify causes and harmonize for classifying stillbirths and early neonatal deaths (death of the neonate within 7 days) data globally (16). Additionally, the ICD-PM was developed to have minimal data requirements, be simple to use, and have fewer clinical details compared to some other recently developed classification systems for perinatal mortality. The ICD-PM code has three main categories: antepartum death (after fetal viability), intrapartum death (during labor and delivery), and early neonatal death (within the first week of birth). Each of these categories represents a distinct period, which helps healthcare professionals understand the timing and potential causes of perinatal mortality (16).

Furthermore, the ICD-PM categories include 6 antepartum causes of death (designated by “A”), 7 intrapartum causes of death (designated by “I”), and 11 neonatal causes of death (designated by “N”). For each cause, the maternal condition contributing to perinatal death is recorded. The existing ICD-10 groups have been reordered and expanded to more accurately represent the spectrum of possible conditions, including a new category for cases where no maternal condition is identified (17). Fetal abnormalities, infection, antepartum hypoxia, fetal growth disorder, placental insufficiency, maternal health conditions, or other complications during pregnancy are the major potential causes of perinatal death during the antepartum period. Intrapartum deaths, which occur during labor and delivery, primarily result from birth asphyxia, birth trauma, low birth weight and prematurity, umbilical cord accidents, or maternal emergencies. Early neonatal deaths are associated with prematurity, congenital anomalies, infections, or other medical conditions (18).

Accurate recording of the cause of death and identification of preventable causes is essential to reducing the global burden of perinatal mortality. Additionally, this helps guide the allocation of limited resources to have the greatest impact on reducing perinatal mortality rates. However, studies using the ICD-PM classification for stillbirths have shown inconsistent results, as highlighted in a previous systematic review that only provided descriptive measures, such as ranges and median values (19). Additionally, discrepancies existed across economic regions, and there are challenges in categorizing a high proportion of antepartum deaths as unspecified causes. Nevertheless, subsequent studies have been published since the review.

In contrast, our review extracted all refined articles on perinatal mortality into Microsoft Excel and organized them accordingly, reporting the data in the form of frequency and percentage for each category of the ICD-PM classification. The data were then tabulated alongside the maternal complications. Furthermore, findings were extracted based on the gestational age used to define perinatal mortality and presented in table format. Additionally, the pooled rates of stillbirth and perinatal mortality were estimated based on the reports of the ICD-PM classification.

## 2 Methods

### 2.1 Data sources and search strategy

This systematic review was reported in accordance with the 2010 Preferred Reporting Items for Systematic Reviews and Meta-Analysis (PRISMA) statement (20). We reviewed the literature for articles, established eligibility criteria, selected relevant studies, critically appraised the included studies, and conducted data analysis and synthesis. The study was registered in Prospero with the number CRD4202452549.

The following electronic databases were systematically searched from 1 January 2016 to 14 March 2024: PubMed via MEDLINE, SCOPUS, Web of Sciences, EMBASE, Cochrane Library, and PROSPERO. We developed a search strategy using Medical Subject Heading (MeSH) terms and keywords. The terms/keywords include “International Classification of Diseases Perinatal Mortality” OR “ICD-PM” AND “mortality, perinatal” OR “perinatal mortality,” OR “perinatal death” OR “stillbirth.” The search strategy is provided in Supplementary File (search strategy). Additionally, manual searching of references of retrieved articles and previous systematic reviews were conducted to include studies missed in database searching.

### 2.2 Eligibility criteria and screening

All original studies or literature published in the English language were included. Articles that used prospective or retrospective cross-sectional, case–control, cohort, and randomized controlled trial designs were eligible. The criteria followed for the inclusion of the studies were causes reported using ICD-10 and studies focused on stillbirth (from 20 weeks of gestation until delivery) and early/late neonatal deaths (deaths during the first 7/28 days of life from birth). Perinatal mortality assessed with other ICD codes or in combination with earlier versions of ICD were not considered in this review. Case reports, case series, and commentaries were also excluded.

After excluding duplicates in EndNote software, all articles searched from the databases were exported to Covidence, a web-based tool for article screening. Two investigators (HK, MMR and NBY) independently screened articles based on their titles and abstracts. Subsequently, the same investigators conducted full-text screening of the articles retained in the first phase. Disagreements between the two were resolved through a discussion between three investigators (HK, NBY, and EM).

### 2.3 Data extraction and quality appraisal

We developed a data extraction form in Microsoft Excel, the first Microsoft Excel, consisting of the author name, year of publication, country, design, sample size, outcome, estimates, ICD-PM causes, and other relevant study population characteristics. The second Microsoft Excel file consists of the author names listed in the columns and rows containing the number of perinatal mortalities tabulated by maternal causes during the antepartum, intrapartum, and neonatal periods. Finally, after all the data were extracted for the antepartum, intrapartum, and neonatal periods, the maternal and fetal factors for each period were aggregated, and data of frequencies and percentages were presented. Data extraction was conducted independently by two investigators (HK and NBY). In cases of inconsistencies, the issues were resolved through discussion and by involving additional third investigators (EM or MMR).

We assessed the quality of the included studies using appropriate tools for the study designs. The NOS for assessing the quality of non-randomized studies in meta-analyses (21) was used to appraise observational studies. A NOS of 7–9 was considered high quality, 5–6 was moderate, and below 5 was low. Studies that scored low quality were not included in the analysis. Similarly, two investigators (HK and EM) assessed the quality of the included studies, and the disagreement between them was resolved through consensus and the involvement of additional authors. The full quality assessment result of the included studies is provided in Supplementary Table S1.

### 2.4 Analysis

The findings of the review were summarized and synthesized qualitatively and quantitatively. The causes and timing of deaths were described qualitatively and using relevant summary measures of frequency and percentage. The stillbirth rate and perinatal mortality rate were calculated per 1,000 births. Tables were used to summarize ICD-PM mortality using frequencies and percentages. We conducted a meta-analysis of the combined estimates using a random-effects (DerSimonian and Laird) method with an inverse-variance approach, adjusting to the study weights (22). Forest plots were used to present the findings graphically. Statistical heterogeneity between the studies was assessed using I^2^ statistic. The findings of the I^2^-test were classified as having low (25%), moderate (50%), and high (75%) heterogeneity (23). When there was evidence of heterogeneity, subgroup analysis was performed to check effects across different groups. We used Egger’s test and funnel plots to assess publication bias. If bias existed, we used a trim-and-fill analysis. Sensitivity analysis was conducted to check the robustness of the findings. All analyses were performed in STATA version 17.0.

## 3 Results

**Figure 1:**
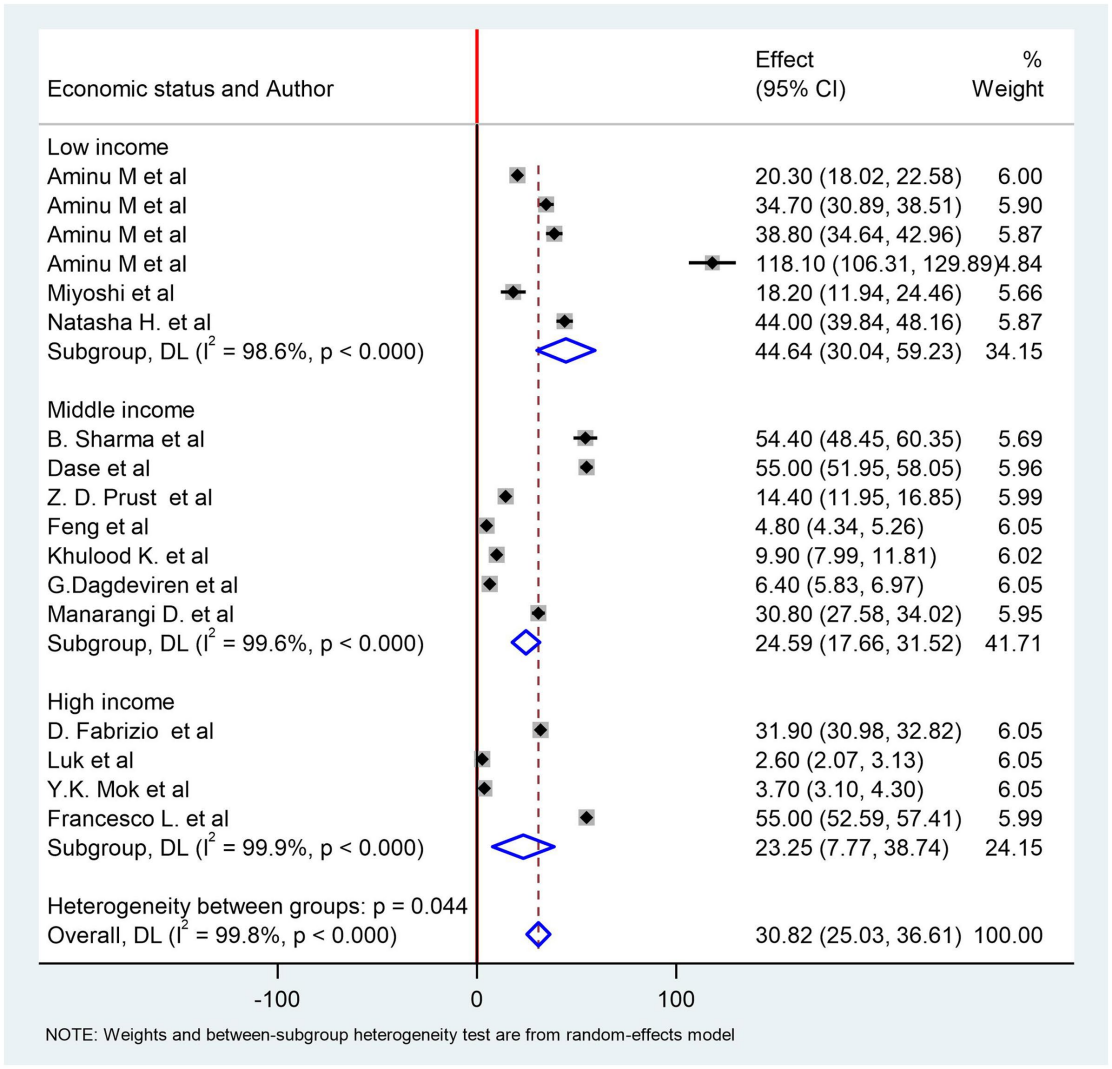
Flow chart of study selection for systematic review and meta-analysis of the global report of ICD-PM classification.

This systematic review and meta-analysis was focused on the ICD-PM classification reports across the globe. A thorough search of electronic databases resulted in the retrieval of 21,644 records. From these records, a careful screening process led to the inclusion of only 23 articles in the ICD-PM classification, and 14 studies were used to estimate the pooled rate of stillbirth and perinatal mortality (Figure 1).

### 3.1 Characteristics of the included studies

This review included 23 studies from various regions around the world. Out of the 23 studies, 17 were conducted in low- and middle-income countries: Zambia (24), Nepal (25), Solomon Island (26), Suriname (27), India (28), Nigeria (29), Pakistan (30), Sri Lanka (31), China (32), Jordan (33), Tanzania (34), Turkey (35, 36), North Macedonia (37), Thailand (38), South Africa (39), and Colombia (40), and one study was conducted at the sub-regional level, which includes four sub-Saharan African countries (Malawi, Zimbabwe, Kenya, and Sierra Leone) (41). The remaining four studies were conducted in high-income countries, particularly in Italy (42, 43) and Hong Kong (44, 45). Additionally, one study was conducted in the United Kingdom and South Africa (46).

The studies were published between 2016 and 2022, with the majority of articles published in 2020. Approximately half of the study designs were retrospective and prospective cohort studies. Regarding the method of assessing perinatal mortality, eight studies have not reported the method of certification of death. The remaining studies stated the certificate of death or reported by ICD-PM-trained professionals. The details are available in Supplementary Table S2.

A total of 48,596 perinatal deaths were reported across the 23 articles reviewed, with approximately 96% (46,816 deaths) classified according to the ICD-PM. Among these deaths, 25,563 (54.6%) were antepartum fetal deaths, reported by all 23 articles, and classified accordingly. Intrapartum deaths were reported by 22 studies, with 6,418 (13.7%) fetal deaths classified based on the ICD-PM system. Additionally, 11 studies included deaths during the neonatal period, with 14,835 (31.7%) neonatal deaths classified according to the ICD-PM coding. A summary of the included articles is available in Table 1 and Supplementary Table S2.

**Table 1:** Characteristics of the included studies.

Author	Publication year	Country	Study design	Sample size	SB rate	PMR	Total death reported	In ICD-PM included
Allanson et al.	2016	UK and SA	Retrospective	NA	NA		9,756	9,748
Priyani AAH et al.	2017	Sri Lanka	Retrospective	NA	NA		291	291
Tina Lavin et al.	2018	South Africa	Not reported	NA	NA		26,810	26,810
Aminu M et al.	2019	Sub-Saharan Malawi Zimbabwe Kenya Sierra Leone	Prospective	14,729 8,847 8,273 2,879	20.3 34.7 38.8 118.1		1,267	968
Miyoshi et al.	2019	Zambia	Retrospective	1754	18.2	42	75	75
Mary S.	2019	Colombia	Cross-sectional	NA	NA		3,901	3,361
B. Sharma et al.	2020	India	Prospective	5,574	54.4		314	314
D. Fabrizio et al.	2020	Italy	Prospective	141,013	31.9		443	432
Dase et al.	2020	Nigeria	Retrospective	21,462	55		1,177	760
T. Wasim et al.	2020	Pakistan	Prospective	11,850	NA	58.2	690	690
Z. D. Prust et al.	2020	Suriname	Cross-sectional	9,089	14.4		113	107
Luk et al.	2020	Hong Kong	Retrospective	34,920	2.6	3.4	119	119
Y.K. Mok et al.	2020	Hong Kong	Retrospective	39,625	3.7		145	135
Khulood K. et al.	2020	Jordan	Prospective	10,328	9.9		102	95
Natasha H. et al.	2021	Tanzania	Prospective	9,333	44	71	744	459
G.Dagdeviren et al.	2021	Turkey	Cross-sectional	74,102	6.4		475	458
Shrestha J et al.	2021	Nepal	Retrospective	NA			461	461
WHO	2021	North Macedonia	Prospective	NA			202	169
Taweevisit et al.	2022	Thailand	Retrospective	NA			330	330
Francesco L. et al.	2022	Italy	Retrospective	34,417	55		191	191
Salih Metin et al.	2022	Turkey	Retrospective	NA	NA		229	229
Manarangi De Silva	2022	Solomon Island	Retrospective	11,056	30.8		341	194
Feng et al.	2024	China	Retrospective	87,588	4.8		420	420
Total							48,596	46,816

NA, not applicable or only included prenatal death reports; SB, stillbirth; SA, South Africa; PMR, perinatal mortality rate; UK, United Kingdom.

### 3.2 Stillbirth and perinatal mortality rate

Out of the 23 studies included in the analysis, only 14 reported the total number of births during the study period, which amounted to a total of 514,989 births. These studies reported stillbirth rates from 17 countries (one sub-regional study that included four Sub-Saharan African countries) (24, 26–29, 32–35, 41–45). From the included studies, nine articles used a gestational age of 28 weeks and above cutoff or a birth weight of more than 1,000 g to define stillbirth (24, 25, 27, 29–31, 34, 39, 41). A study conducted in the United Kingdom and South Africa (46) used a cutoff of 24 and 28 weeks for the two countries, respectively. The remaining 13 studies defined stillbirth as a fetal death starting between 20 and 28 weeks of gestation or weighing 350/500 g and above (26, 28, 32, 33, 35–38, 40, 42–45).

The highest stillbirth rate was reported in a study from Sierra Leone, with a rate of 118/1,000 births (41), while the lowest rate was reported from Hong Kong at 2.6/1,000 births (44). The pooled rate of stillbirth from 14 studies in the global report of ICD-PM classification is 31/1,000 births (95%CI: 25.03, 36.61). We stratified the studies based on the economic classification of countries. The pooled rate of stillbirths in high-income countries using four studies (42–45) was 23/1,000 births (95%CI: 7.77, 38.74). The level of heterogeneity was high, as evidenced by an I^2^ value of 97.9% (*p* < 0.0001).

**Figure 2:**
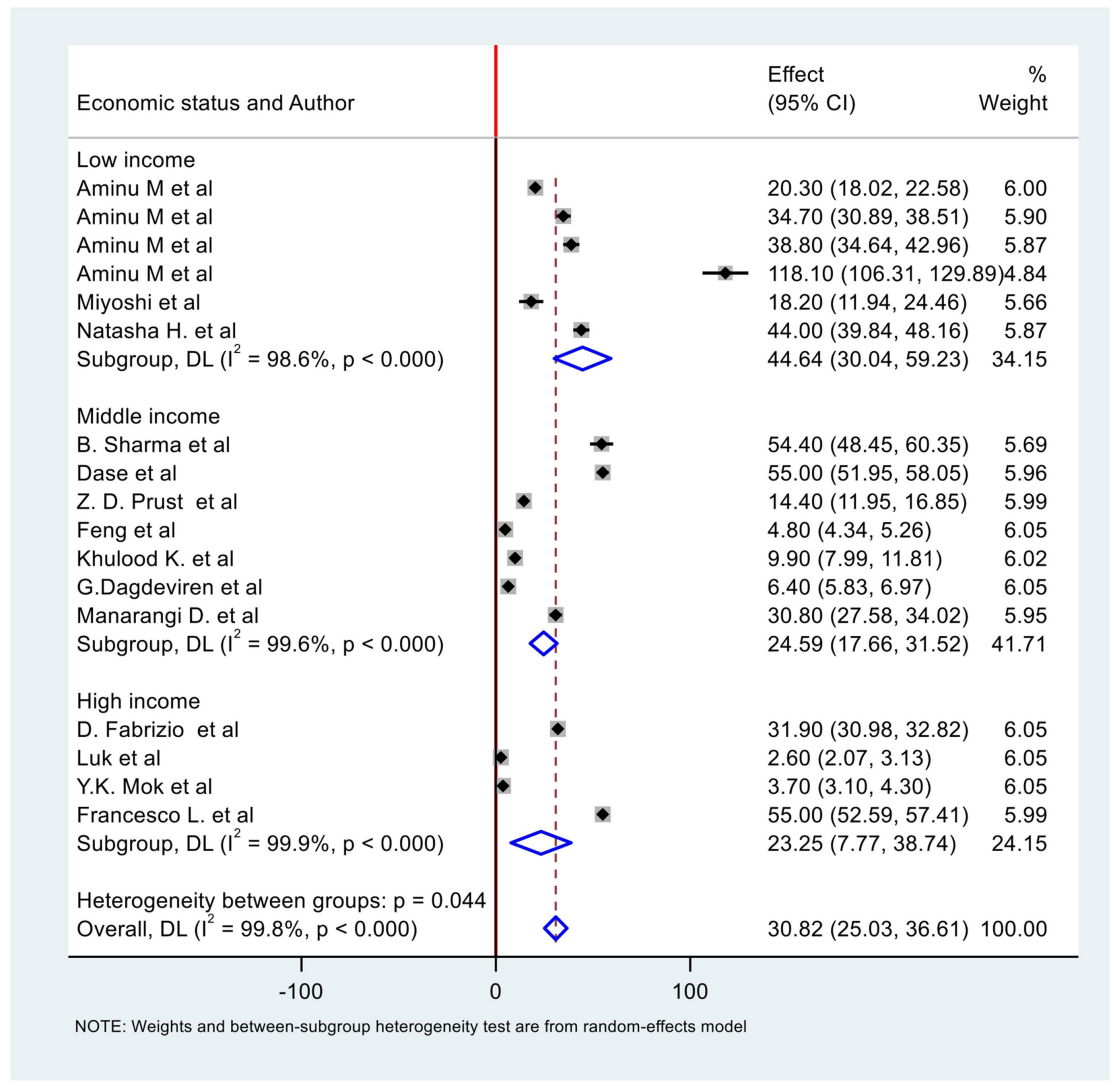
Forest plot of stillbirth rate from studies used ICD-PM classification.

Moreover, the pooled rate of stillbirth in middle-income countries using seven studies (26–29, 32, 33, 35) was 25/1,000 births (95% CI: 17.66, 31.52) with a high level of heterogeneity. In low-income countries, the pooled rate of stillbirth (24, 34, 41) was found to be 45/1,000 births (95%CI: 30.04, 59.23) (Figure 2). Similarly, the level of heterogeneity was high. We conducted Egger’s test to examine the presence of publication bias and obtained a *p*-value of 1. This result indicates that there is no statistically significant evidence of publication bias in the included studies. Egger’s test result is available in Supplementary File under the subtitle of Egger’s test. The funnel plots are also available in Supplementary Figure S1.

Four studies (24, 30, 34, 44) reported perinatal deaths, with one study by Wasim et al. in Pakistan (30) including deaths up to 7 days or early neonatal deaths only, while the other three studies reported deaths within the neonatal period (28 days). The highest perinatal death rate was reported in Tanzania at 71/1,000 live births (34), and the lowest rate was reported in Hong Kong at 3.4/1,000 births (44). Further details of the studies’ reports are found in Table 1 and Supplementary Table S2.

**Figure 3:**
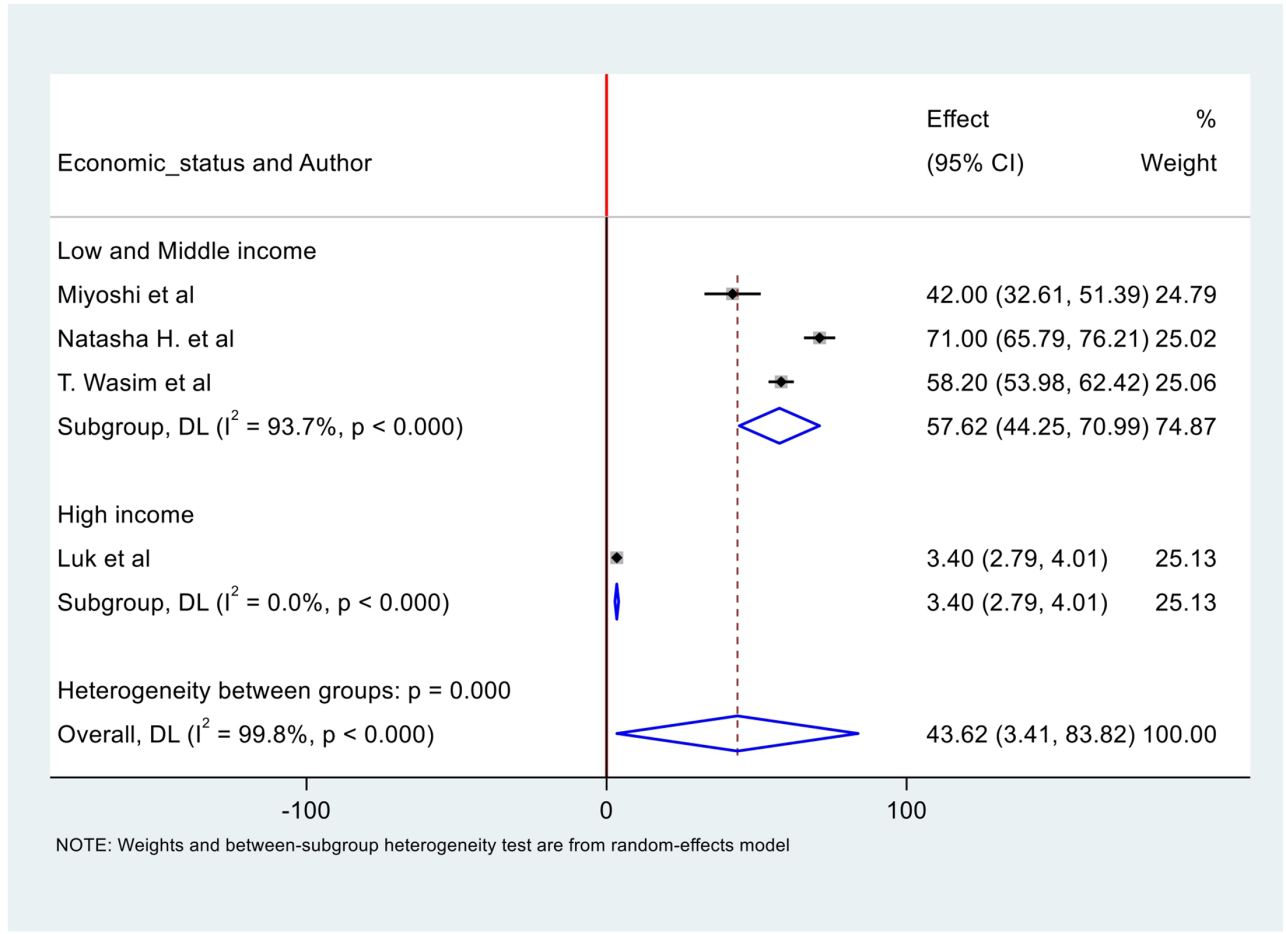
Forest plot of perinatal mortality rate from studies used ICD-PM classification.

The pooled rate of perinatal mortality from four studies (24, 30, 34, 44) was 44/1,000 births. The level of heterogeneity was high (*p* < 0.0001). When we exclude the study by Luk et al., which was conducted in a high-income country (44), the pooled perinatal mortality rate is approximately 58/1,000 live births (Figure 3). Egger’s test was conducted and the result revealed the absence of publication bias. The result is available in Supplementary File in Egger’s test subsection. The funnel plots are available in Supplementary Figure S2.

### 3.3 ICD-PM classification

A total of 23 studies were included in this review, which reported on stillbirth or perinatal mortality. However, three of Priyani et al. (31), Shattnawi et al. (33), and Lupariello et al. (43) these studies did not provide tabulation with maternal conditions (details of maternal conditions (M1–M5) are available in Supplementary Table S3). From these, Francesco et al. (43) reported maternal complications, including antepartum and intrapartum maternal complications. We excluded the maternal complications for this review (43). Khulood et al. (33) reported the fetal and maternal causes separately. Priyani et al. (31) reported the fetal and neonatal causes of death but did not tabulate the maternal causes, including the antepartum, intrapartum, and neonatal classifications.

For a detailed understanding of the causes of death, the reviewed studies were classified into three categories. The first category, which comprised all deaths regardless of gestational age, was used to define stillbirth or PM. The second category includes 14 studies that used to define stillbirth or PM, after 20 weeks of gestation. Within this category, three studies that reported on pregnancies after 20 weeks are from the Solomon Islands (26), China (32), and Jordan (33). Six studies that reported on pregnancies after 22 weeks were from India (28), North Macedonia (37), Thailand (38), Colombia (40), and Italy (42, 43). Additionally, four studies reported on pregnancies after 24 weeks of gestation were from Turkey (35, 36), the United Kingdom (46), and Hong Kong (44, 45). Two studies from Thailand (38) and Colombia (40) had two categories: 22–28 and 28 and above. The third category consisted of 10 studies, all of which were conducted in low- and middle-income countries and defined perinatal mortality after 28 weeks and above gestation.

### 3.4 Antepartum death for all studies

Table 2 presents the results of the included studies in the review, along with the classification of antenatal deaths and maternal tabulation. Antepartum deaths were reported in all (24–46) articles, with a total of 25,563 recorded deaths. The most common cause for antepartum stillbirths was unspecified antepartum death 14,8,872 (58.2%), and more than half of these deaths were accompanied by M5 (no maternal condition/healthy mother), followed by other (A4) 3,362 (13.1%) and by fetal growth disorders 2,804 (11%).

**Table 2:** Causes perinatal death according to ICD-PM of all studies.

Maternal condition	M1:Complication of placenta, cord, membrane	M2: Maternal complication of pregnancy	M3:Other complications of labor and delivery	M4:Maternal surgical and medical conditions	M5:No maternal conditions	Three studies (31, 33, 43) *	Total (%)
Antepartum death							25,563
A1: Congential malformations and chromosomal abnormalities	168	440	19	156	1,105	61	1949 (7.6)
A2: Infection	162	24	3	369	72	32	662 (2.5)
A3: Antepartum hypoxia	817	123	30	483	251	195	1899 (7.4)
A4: Other specified antepartum disorder	2,452	55	23	652	135	45	3,362 (13.1)
A5: Disorder related to fetal growth	559	637	29	739	813	27	2,804 (11)
A6: Antepartum death unspecified cause	1,148	252	43	5,102	8,241	101	14,887 (58.2)
Khulood K. et al.	19	9	2	4	44	–	–
Total (%) M1-M5 = 25,180	5,325 (21.1)	1,540(6.1)	149(25.4)	7,505(20.7)	10,661(16.8)		
Intrapartum death							6,418
I1: Congenital malformation and chromosomal abnormalities	9	27	114	41	287	9	487 (7.6)
I2: Birth trauma	2	0	6	8	4	0	20 (0.3)
I3: Acute intrapartum event	1,246	115	1,201	645	494	11	3,712 (57.8)
I4: Infection	28	8	7	51	6	0	100 (1.5)
I5: Other specified intrapartum disorder	352	57	26	87	24	4	550 (8.6)
I6: Disorder related to fetal growth	130	33	65	75	89	1	393 (6.1)
I7: Intrapartum unspecified cause	290	67	212	412	170	5	1,156 (18)
Khulood K. et al.	4				8		
Total (%) M1-M5 = 6,400	2061 (32.2)	307 (4.8)	1,631 (25.5)	1,329 (20.6)	1,082 (16.9)		
Neonatal Death							14,835
N1: Congenital malformations, deformations, and chromosomal abnormalities	94	271	10	196	1903	38	2,512 (16.9)
N2: Disordersrelatedtofetal growth	23	2	3	64	89	3	184 (1.2)
N3: Birth trauma	0	0	1	3	10	0	14 (0.09)
N4: Complications of intrapartum event	288	15	1707	360	151	11	2,532 (17)
N5: Convulsions and disorders of cerebral status	20	3	22	26	91	0	162 (1.1)
N6: Infections	99	39	42	212	425	1	818 (5.5)
N7: Respiratory and cardiovascular disorders	90	72	81	832	1,311	6	2,392 (16.1)
N8: Otherneonatalconditions	358	14	7	94	245	2	720 (4.8)
N9: Low birth weight and prematurity	502	456	2,319	477	313	24	4,091 (27.6)
N10: Miscellaneous	14	8	0	97	124	0	243 (1.6)
N11: Neonataldeathof unspecified cause	293	71	19	113	670	1	1,167 (7.8)
Total (%) M1-M5 = 14,749	1,781 (12)	951 (6.4)	4,211 (28.5)	2,474(16.8)	5,332 (36.2)		

* Studies reported based on ICD-PM classification but do not tabulated with maternal condition.

### 3.5 Intrapartum death for all studies

Except for the study from China (32), the remaining 22 studies (24–31, 33–46) reported a total of 6,418 intrapartum deaths classified according to the ICD-PM coding. The leading causes of perinatal mortality during the intrapartum period were acute intrapartum events (I3), accounting for 3,712 cases (58.1%), and unspecified causes, with 1,156 cases (17.9%). Among these, maternal tabulation was also available in 6,400 cases. The leading maternal causes of perinatal mortality were complications of the placenta, cord, and membranes (M1) and complications of labor and delivery (M3) (Table 2).

### 3.6 Neonatal deaths for all studies

Eleven studies (24, 25, 30, 31, 34, 36, 37, 39, 40, 44, 46) reported 14,835 neonatal deaths according to ICD-PM coding. However, there are discrepancies in the reporting of perinatal mortality. Six studies (24, 34, 36, 40, 44, 46) that reported neonatal deaths include the first 28 days of life, and five studies (25, 30, 31, 37, 39) considered only early neonatal deaths. The most common cause of neonatal death was N9, preterm, or low birth weight (57.6%), followed by N4, complications of an intrapartum event (17%); all of these conditions were associated with maternal complications of labor and delivery. The third cause of death was congenital malformations (16.9%) (Table 2).

### 3.7 Perinatal mortality defined after 20 weeks

Table 3 shows findings based on studies that defined perinatal mortality after 20 weeks of gestation or (350/500 g and above for unknown gestational age) until 7/28 days of the postpartum period. However, studies that defined perinatal mortality after 28 weeks of gestation were excluded. Fourteen studies were included and the studies were from Solomon Island (26), India (28), China (32), Jordan (33), Turkey (35, 36), North Macedonia (37), Thailand (38), Colombia (40), Italy (42, 43), Hong Kong (44, 45), and the United Kingdom (46). In two studies, the perinatal mortalities were not tabulated with maternal conditions and reported the antepartum and intrapartum causes of death (33, 43).

**Table 3:** Causes perinatal death according to ICD-PM for perinatal mortality define gestational age after 20 weeks.

Maternal condition	M1:Complication of placenta, cord, membrane	M2: Maternal complication of pregnancy	M3:Other complications of labor and delivery	M4:Maternal surgical and medical conditions	M5:No maternal conditions	Two authors (33, 43)*	Total (%)
Antepartum death							8,094
A1: Congential malformations and chromosomal abnormalities	161	427	19	72	736	33	1,448 (17.9)
A2: Infection	75	21	2	34	20	24	176 (2.2)
A3: Antepartum hypoxia	673	89	25	207	219	112	1,325 (16.4)
A4: Other specified antepartum disorder	102	39	23	53	129	10	365 (4.4)
A5: Disorder related to fetal growth	407	92	29	275	410	17	1,230 (15.2)
A6: Antepartum death unspecified cause	798	154	42	339	2,159	67	3,559 (44)
Khulood K. et al.	19	9	2	4	44		
Total (%) M1-M5 = 7,909	2,235 (28.2)	831 (10.5)	142 (1.7)	984 (12.4)	3,717 (47)		
Intrapartum death							1,267
I1: Congenital malformation and chromosomal abnormalities	6	16	92	8	99	4	225 (17.6)
I2: Birth trauma	2	0	6	8	4	0	20 (1.6)
I3: Acute intrapartum event	244	47	74	42	195	10	612 (47.9)
I4: Infection	16	3	0	5	5	0	29 (5.3)
I5: Other specified intrapartum disorder	2	5	26	9	23	4	69 (5.3)
I6: Disorder related to fetal growth	27	22	38	23	55	0	165 (12.9)
I7: Intrapartum unspecified cause	56	12	14	18	53	5	158 (12.4)
Khulood K. et al.	4				8		
Total (%) M1-M5 = 1,276	357 (28.2)	105 (8.3)	250 (19.7)	113 (8.9)	442 (34.9)		
Neonatal death							6,147
N1: Congenital malformations, deformations, and chromosomal abnormalities	85	259	7	55	1,276		1,682 (27.3)
N2: Disordersrelatedtofetal growth	4	2	3	4	10		23 (0.37)
N3: Birth trauma	0	0	1	3	10		14 (0.2)
N4: Complications of intrapartum event	60	8	12	8	97		185 (3)
N5: Convulsions and disorders of cerebral status	9	0	4	1	40		54 (0.9)
N6: Infections	28	8	3	15	331		385 (6.2)
N7: Respiratoryand cardiovascular disorders	50	45	15	39	564		713 (11.6)
N8: Other neonatal conditions	21	10	4	10	241		286 (4.6)
N9: Lowbirthweight and prematurity	409	153	730	74	231		1,597 (26)
N10: Miscellaneous	8	3	0	0	22		33 (0.05)
N11: Neonataldeathof unspecified cause	294	72	17	110	682		1,175 (19.1)
Total (%) M1-M5 = 6,147	968 (15.7)	560 (9.1)	796 (12.9)	319 (5.2)	3,504 (57)		

* Studies reported based on ICD-PM classification but do not tabulated with maternal condition.

### 3.8 Antepartum death after 20 weeks of gestation

In this gestational age category, 8,094 antepartum deaths were classified according to ICD-PM classification from 14 studies (26, 28, 32, 33, 35–38, 40, 42–46). Congenital malformation and chromosomal abnormalities (A1) 1,448 (17.9%) and antepartum hypoxia (A3) 1,325 (16.4%) were the leading causes of perinatal mortality during the antepartum period. From these, 7,909 maternal complications were tabulated with perinatal mortality. The leading tabulated causes are complications of placenta, cord, and membrane (M1) 2,235 (28.2%) and no maternal condition (M5) 3717 (47%).

### 3.9 Intrapartum deaths after 20 weeks of gestation

Except for the study from China (32), the aforementioned 13 studies (26, 28, 32, 33, 35–38, 40, 42–46) reported a total of 1,278 intrapartum deaths, which were classified according to the ICD-PM. Among these deaths, acute intrapartum events (I3) accounted for 612 cases (47.9%), and congenital, malformations, and chromosomal abnormalities (I1) were responsible for 225 cases (17.6%). From 1,276 cases classified as intrapartum maternal causes, complications of placenta, cord, and membrane were identified as the cause for 357 deaths (28.2%), and no maternal conditions were identified as the cause for 442 deaths (34.9%).

### 3.10 Neonatal deaths after 20 weeks of gestation

In five studies (36, 37, 40, 44, 46), 1,914 neonatal deaths were reported using the ICD-PM classification. Except for a study conducted by the WHO in North Macedonia (37), which included early neonatal death, the remaining studies focussed on the first 28 days of life. The leading causes of neonatal deaths were congenital malformations, and chromosomal abnormalities (N1) with 1,682 cases (27.3%), respiratory and cardiovascular disorders (N7) with 713 cases (11.6%), and low birth weight and prematurity (N9) with 1,597 cases (26%).

### 3.11 Perinatal mortality after 28 weeks

Twelve studies (24, 25, 27, 29–31, 34, 38–41, 46) defined perinatal mortality as the death of a fetus after 28 weeks of gestation and until 7/28 days of postpartum day. However, studies from Thailand (38) define perinatal mortality (PM) after 22 weeks of gestation. The results were categorized into two groups: 22–28 weeks (less than 1,000 g) and 28 weeks or more (1,000 g and above). These findings were presented separately, and the results for gestational age of 28 weeks or more, or birth weight of 1,000 g and above, were included in this category (Table 4).

**Table 4:** Causes perinatal death according to ICD-PM for perinatal mortality define gestational age after 28 weeks.

Maternal condition	M1:Complication of placenta, cord, membrane	M2: Maternal complication of pregnancy	M3:Other complications of labor and delivery	M4:Maternal surgical and medical conditions	M5:No maternal conditions	One author (31)*	Total (%)
Antepartum death							18,343
A1: Congential malformations and chromosomal abnormalities	19	17	5	86	483	28	638 (3.5)
A2: Infection	101	7	2	346	57	8	521 (2.8)
A3: Antepartum hypoxia	386	59	20	334	175	83	1,057 (5.8)
A4: Other specified antepartum disorder	2,363	20	13	612	30	35	3,073 (16.7)
A5: Disorder related to fetal growth	182	552	5	480	438	10	1,667 (9.1)
A6: Antepartum death unspecified cause	358	106	22	4,770	6,097	34	11,387(62.1)
Total (%) M1-M5 = 18,145	3,409 (20.1)	761 (5.6)	67 (0.3)	6,628 (31.1)	7,280 (42.9)		
Intrapartum death							5,462
I1: Congenital malformation and chromosomal abnormalities	3	16	84	33	245	5	386 (7.1)
I2: Birth trauma	0	0	1	0	0	0	6 (0.002)
I3: Acute intrapartum event	1,042	81	1,146	611	341	1	3,222 (59)
I4: Infection	18	6	7	48	4	0	83 (1.5)
I5: Other specified intrapartum disorder	350	54	23	84	6	0	517 (9.4)
I6: Disorder related to fetal growth	107	12	32	53	37	1	242 (4.4)
I7: Intrapartum unspecified cause	234	56	207	394	120	0	1,011 (18.5)
Total (%) M1-M5 = 5,455	1754 (32.2)	225 (4.1)	1,500 (27.5)	1,223 (22.4)	753 (13.8)	–	–
Neonatal death							9,901
N1: Congenital malformations, deformations, and chromosomal abnormalities	13	18	5	144	1,080	38	1,298 (13.1)
N2: Disorders related to fetal growth	19	0	0	60	82	3	164 (1.6)
N3: Birth trauma	0	0	0	0	2	0	2 (0.02)
N4: Complications of intrapartum event	250	11	1701	356	93	11	2,422 (24.5)
N5: Convulsions and disorders of cerebral status	13	3	20	25	71	0	132 (1.3)
N6: Infections	82	37	40	206	300	1	666 (5.2)
N7: Respiratoryand cardiovascular disorders	44	34	72	797	969	6	1,922 (19.4)
N8: Other neonatal conditions	341	4	5	85	102	2	539 (5.4)
N9: Low birth weight and prematurity	100	307	1,591	408	98	24	2,528 (25.5)
N10: Miscellaneous	5	3	0	96	75	0	179 (1.8)
N11: Neonatal death of unspecified cause	0	1	3	5	39	1	49 (0.5)
Total (%) M1-M5 = 9,815	867 (8.8)	418 (4.2)	3,437 (35)	2,182 (22.2)	2,911 (29.6)	--	--

* Studies reported based on ICD-PM classification but do not tabulated with maternal condition.

### 3.12 Antepartum death after 28 weeks

Twelve studies (24, 25, 27, 29–31, 34, 38–41, 46) included 18,343 antepartum deaths in ICD-PM. The leading cause of antepartum death was categorized under antepartum death, unspecified cause (A6) 11,387 (62%), which is frequently associated with maternal surgical and medical conditions. The maternal tabulation revealed that three-fourths of the causes of antepartum death were no maternal conditions (M5) and maternal surgical and medical conditions (M4) (Table 4).

### 3.13 Intrapartum death after 28 weeks

Twelve studies (24, 25, 27, 29–31, 34, 38–41, 46) reported and classified 5,462 intrapartum deaths according to the ICD-PM classification. The leading cause of death was an acute intrapartum event (I3), 3,222 (59%). Furthermore, nearly 60% of maternal causes of death were complications of the placenta, cord, membrane (M1), and other complications of labor and delivery (M3) (Table 4).

### 3.14 Neonatal death after 28 weeks

Eight studies (24, 30, 31, 33, 34, 40, 46, 47) reported 9,901 neonatal deaths according to ICD-PM coding. Four studies (24, 34, 40, 46) included both early and late neonatal death, and three studies (30, 33, 47) reported only early neonatal death. Half of the neonatal deaths were caused by complications of the intrapartum event (N4) 2422 (24.5) and low birth weight and prematurity (N9) 2528(25.5%) (Table 4).

### 3.15 Sensitivity analysis

To assess the influence of individual studies on the overall stillbirth rate, a leave-one-out sensitivity analysis was conducted. The results of the sensitivity analysis indicated that the pooled rates were not influenced by any single study. The detailed results of this analysis are found in Supplementary Figure S3 and Supplementary Table S4 in the section titled Sensitivity Analysis.

## 4 Discussion

### 4.1 Main findings

This review was conducted to determine the rate of perinatal mortality and identify causes of perinatal mortality according to ICD-PM based on existing global reports. More than half a million births were reported from 14 studies and the pooled stillbirth rate was 31/1,000 births globally. However, the pooled stillbirth rate in low-income countries is unacceptably high. From 23 studies, 46,816 perinatal mortalities were classified according to ICD-PM. The most commonly identified causes for antepartum deaths coding on other specified antepartum disorders (A4: Vasa previa, ruptured cord, twin-twin transfusion etc.) (13%). Moreover, regardless of gestational age used to define stillbirth or PM this review revealed 17.9% of neonatal deaths were caused by congenital malformations and chromosomal abnormalities.

Acute intrapartum events (I3) accounted for the largest proportion of intrapartum deaths for all classifications applied in this review. Deaths without specific fetal cause occur in 2 out of 10 intrapartum deaths among studies that defined perinatal mortality above 28 weeks of gestation. Whereas, studies considered above 20 weeks or conducted in high- or middle-income countries reported 1 out of 20 perinatal mortalities during the intrapartum period. The leading cause of perinatal mortality during the neonatal period for gestational age 20 weeks and above was congenital malformation, deformation, and chromosomal abnormalities (27.3%). However, low birth weight and prematurity (25.5%) were reported as common causes of perinatal death defined after 28 weeks.

For associated maternal conditions, the complications of placenta, cord, and membranes (M1) (28.2%) and maternal surgical and medical conditions (M4) (31.1%) categories were the most common category assigned for antepartum deaths for perinatal death defined above 20 and 28 weeks, respectively. Complications of labor and delivery (M3) (27.5) accounted for the highest proportion of intrapartum deaths for gestational age above 28 weeks categories. In the studies defining perinatal mortality as the death of a fetus after 20 weeks of gestation, 57% of neonatal deaths were not associated with maternal conditions, however, neonatal deaths were commonly associated with congenital malformation and chromosomal abnormalities.

### 4.2 Comparison with existing literature

This review revealed a pooled stillbirth rate of 45/1,000 births using ICD-PM classification reports from low-income countries. A meta-analysis study conducted using demographic and health survey data in Sub-Saharan Africa showed a stillbirth rate of 34/1,000 births (4), which is lower than the present finding. This difference might be explained by the relatively better reporting systems in hospital-based registries compared to a population-based survey (48). The stillbirth rate in low-income countries remains quite high, and the countries in the region are unlikely to achieve the Sustainable Development Goal target of 12 stillbirths per 1,000 births by 2030 (8, 49).

The stillbirth rate reflects the quality of healthcare that women receive during the perinatal period (50). According to the WHO, deficiencies in antenatal care contribute to increased stillbirth rates (51). Furthermore, research showed that high-quality antenatal care and the active involvement of healthcare providers in educating mothers about pregnancy danger signs can reduce stillbirths (52). Investing in the healthcare system and providing good-quality and timely maternal services may prevent significant rates of stillbirths (53). Therefore, to achieve the Sustainable Development Goal target 2030, an evaluation of perinatal mortality policies and strategies might be needed in countries with a high level of stillbirth or perinatal mortality.

Our review using the ICD-PM coding system on a global report revealed that the majority of perinatal mortality occurs during the antepartum period. Nearly half of the antepartum deaths were reported to be associated with maternal complications of the placenta, cord, surgical, or medical conditions. Additionally, the majority of antepartum deaths were coded under A6, which is death with an unspecified cause but mainly associated with the complication of cord, placenta, and membrane, as well as surgical and medical conditions of the women. Likewise, a systematic review and meta-analysis study conducted in South Asia and Sub-Saharan Africa revealed stillbirth is associated with premature rupture of the membrane, diabetes mellitus, hypertension, advanced maternal age, antepartum hemorrhage, and anemia (54, 55).

Acute intrapartum events, or intrauterine hypoxia, were the leading causes of intrapartum death and were commonly associated with the maternal condition of placenta, cord, and membrane complications. A systematic review conducted in low-income countries also revealed similar findings: placental causes (7.4–42%), asphyxia and birth trauma (3.1–25%), umbilical problems (2.9–33.3%), and amniotic and uterine factors (6.5–10.7%) were leading causes of perinatal mortality (56). Additionally, a systematic review revealed that uterine rupture after prior myomectomy (surgical removal of uterine fibroids) occurred mainly earlier than 36 weeks of gestation and the onset of labor (57).

Our review of ICD-PM coding and different studies highlights the already-established importance of investment in antenatal care to reduce perinatal mortality (4). However, the effect of existing implementation programs in perinatal care services might need to be evaluated on perinatal mortality reduction. For instance, in 2016, the WHO recommended increased antenatal care contacts in the third trimester (58). In response to these recommendations, countries such as Ethiopia and South Africa incorporated their national guidelines (47, 58).

Additionally, various interventions have a clear benefit in reducing stillbirth rates. These include nutritional interventions, midwife-led models of care, trained traditional birth attendants (reducing stillbirths by 31% in low- and middle-income countries), insecticide-treated anti-malarial nets (reducing fetal loss by 33%), smoking cessation, support for women at risk of low birth weight, carrying personal case notes, diuretics, nitric oxide, progesterone, antioxidants for preventing preeclampsia, altered dietary salt, screening for gestational diabetes and thyroid dysfunction, diet and exercise for preventing gestational diabetes, ultrasound for fetal assessment in early and late pregnancy, fetal movement counting, fetal and umbilical Doppler ultrasound, uteroplacental Doppler ultrasound, antenatal cardiotocography, and symphysial fundal height measurement for detecting abnormal fetal growth (59).

A commonly cited cause of perinatal mortality is prematurity and prematurity-related (54, 60, 61). However, simply identifying that prematurity is an important contributor to deaths gives no information regarding the optimal timing for interventions (62). From the ICD-PM classification, we see that approximately 16% of the total perinatal mortality (A5, I6, and N9) was due to prematurity, and 63.1% (29,254/46,329) of these deaths were also related to a maternal complication. This information is vital to public health experts and policymakers in targeting interventions; a heightened awareness of the causes of such deaths allows a focus on preterm-related issues, underscoring that both obstetric and neonatal interventions are required.

Nearly 11% of the overall causes of perinatal mortality were due to congenital deformations, malformations, and chromosomal abnormalities. These causes were more commonly noted in perinatal deaths occurring after 20 weeks of gestation (21.6%) or in studies conducted in high- or middle-upper-income countries, compared to deaths occurring at 28 weeks and above or in low-income countries (6.8% 6). This might be due to the detection of chromosomal abnormalities using fetal autopsy and genetic evaluation in studies conducted in high-income countries (44). Likewise, a systematic review conducted in low-income countries showed congenital anomalies accounted for 2.1–33.3% of stillbirths (56). Furthermore, a meta-analysis study conducted in Africa also revealed anencephaly alone constitutes 694,857 from 4,963,266 births (63).

Additionally, a meta-analysis study conducted in Denmark also showed congenital malformations were the leading causes of stillbirth (64). The national screening program for congenital malformation detected many severe malformations using ultrasonography (64). This implies the healthcare system might be delayed in addressing preventable stillbirth causes with nutritional supplementation of folic acid. Therefore, the integration of early detection of congenital abnormalities and folic supplementation into routine antenatal care is essential in the reduction of perinatal mortality.

The implementation of ICD-PM coding with simplified training can greatly facilitate its adoption in low-resource settings, enabling better tracking and analysis of maternal and perinatal outcomes. This approach aligns to achieve sustainable development targets (47). Therefore, we strongly recommend that low-resource settings utilize the ICD-PM classification system. By adopting this coding system on an international scale, a consistent and globally recognized classification of perinatal deaths can be established, enabling policymakers, clinicians, and researchers to access comparable data and make informed decisions.

This review represents the first global report in the ICD-PM classification, categorizing ICD-PM reports based on gestational age definitions used in the studies. Furthermore, the rates of stillbirth and perinatal death were pooled from available hospital-based reports using ICD-PM coding, which is considered reliable and reflective of the actual scope of the issue. However, a limitation of this study is that we did not assess the challenges associated with implementing ICD-PM coding. Additionally, this review included studies that used retrospective data, which opens the possibility of biases and potentially limits the generalizability of the findings. Therefore, future studies should focus on addressing these issues to enhance the accuracy of the ICD-PM classification.

## 5 Conclusion and recommendations

In conclusion, this review highlights the high global stillbirth rate, particularly in low-income countries, and antepartum deaths, which identify the leading causes of perinatal mortality based on the ICD-PM classification. Antepartum deaths are commonly attributed to disorders related to fetal growth and other antepartum-unspecified causes. Furthermore, the analysis suggests that a substantial number of deaths are classified as antepartum death (58%), intrapartum death (18%), and neonatal deaths (7.8%) without a specific fetal/neonatal cause. The number increased when studies were only conducted in low- or middle-income countries. In every 10 antepartum deaths, 6 were categorized under antepartum death with unspecified cause. Thus emphasizing the importance of further investigation to enhance understanding and reduce unexplained perianal mortalities.

Intrapartum deaths are predominantly categorized as acute intrapartum events, while neonatal deaths are caused by respiratory and cardiovascular disorders, as well as low birth weight and prematurity. Congenital malformations and chromosomal abnormalities also significantly contribute to perinatal mortalities. Therefore, it is vital to implement a comprehensive approach to address fetal growth disorders, including enhanced prenatal screening, targeted interventions for placental and maternal conditions, improved detection and management of congenital anomalies, strengthened maternal-fetal medicine expertise, and addressing social determinants of health. These measures aim to prevent preterm birth and ensure access to specialized neonatal intensive care services, ultimately optimizing fetal development and preventing perinatal death.

Accurate classification and reporting of perinatal mortality according to the ICD-PM system are crucial for understanding the patterns and addressing the causes of perinatal deaths, ultimately leading to improved maternal and perinatal outcomes worldwide. International organizations can promote the adoption of the ICD-PM classification system globally, enabling standardized reporting and data comparability for monitoring perinatal mortality trends. Further research should be conducted to evaluate the existing policies on perinatal care and the effectiveness of interventions in low-income countries.

## Data Availability

The original contributions presented in the study are included in the article/Supplementary material, further inquiries can be directed to the corresponding author.
