# Supplementary material for: A systematic review and meta-analysis of the globally reported International Classification of Diseases to Perinatal Mortality (ICD-PM)
Source: Front Med (Lausanne). 2024 Sep 23;11:1434380. doi: 10.3389/fmed.2024.1434380 (PMC11457888; doi:10.3389/fmed.2024.1434380)
Supplement: Supplementary file 1 [file Data_Sheet_1.docx]

**Supplementary file**

**Search strategy**

The search was performed using Medical Subject Heading (MeSH) terms associated with ICD-PM. The MeSH terms allow us to identify relevant research articles. We conducted the search by utilizing Boolean operators "AND" and "OR," both independently and in combinations. The search conducted by using MeSH terms related with perinatal mortality. The search terms was ((International Classification of Diseases Perinatal Mortality) OR (ICD-PM)) AND (perinatal mortality)) OR (mortality, perinatal)) OR (perinatal death)) OR (perinatal death)) OR (perinatal mortality)) OR (stillbirth)) OR (Late fetal death)).

Table S1: Quality assessment of the studies included in the systematic review and meta-analysis

| No | Author | Publication Year | Quality of level |
| --- | --- | --- | --- |
|  | Allanson et al | 2016 | 7 (High) |
|  | Priyani AAH et al | 2017 | 6 (Moderate) |
|  | Tina Lavin et al | 2018 | 7 (High) |
|  | Aminu et al | 2019 | 8 (High) |
|  | Miyoshi et al | 2019 | 7 (High) |
|  | Mary S. et al | 2019 | 7 (High) |
|  | B. Sharma et al | 2020 | 8 (High) |
|  | D. Fabrizio et al | 2020 | 7 (High) |
|  | Dase et al | 2020 | 6 (Moderate) |
|  | T. Wasim et al | 2020 | 8 (High) |
|  | Z. D. Prust et al | 2020 | 7 (High) |
|  | Luk et al | 2020 | 7 (Moderate) |
|  | Feng et al | 2020 | 7 (Moderate) |
|  | Y.K. Mok et al | 2020 | 6 (Moderate) |
|  | Khulood K. et al | 2020 | 7 (High) |
|  | Momina Zulfeen et al | 2020 | 5 (Low) |
|  | Natasha H. et al | 2021 | 6 (Moderate) |
|  | G.Dagdeviren et al | 2021 | 7 (High) |
|  | Shrestha J et al | 2021 | 7 (High) |
|  | WHO | 2021 | 7 (High) |
|  | Taweevisit et al | 2022 | 7 (High) |
|  | Francesco L. et al | 2022 | 6 (Moderate) |
|  | Salih Metin et al | 2022 | 6 (Moderate) |
|  | Manarangi D. et al | 2022 | 7 (High) |

Table S2: Characteristics of included studies

| Author | Year of publication | study country | Sample size | Study design | SB  rate | PNM rate | Antepartum death | Intrapartum death | Neonatal death | Total  Death | Not included in ICD-PM | ICD-PM  Included | Method of assessing death | GA used to define PM |
| --- | --- | --- | --- | --- | --- | --- | --- | --- | --- | --- | --- | --- | --- | --- |
| Allanson et al | 2016 | UK and SA | NA | Retrospective | NA |  | 4717 | 530 | 4501 | 9756 | 8 | 9748 | Certifier | 28 and above/Birth weight of ≥1000 grams for SA/UK 24 weeks |
| Priyani AAH et al | 2017 | Sri Lanka | NA | Retrospective | NA |  | 198 | 7 | 86 | 291 | 0 | 291 | Not reported | 28 and above weeks of gestation |
| Tina Lavin et al | 2018 | South Africa | NA | Retrospective | NA |  | 15619 | 3725 | 7466 | 26810 | 0 | 26810 | Audit system | >> |
| Aminu M et al | 2019  2 | Sub-Sahara Malawi  Zimbabwe  Kenya  Sierra Leone | 14729  8847  8273  2879 | Prospective | 20.3  34.7  38.8  118 |  | 325 | 643 | Not included | 1267 | 299 | 968 | Trained team | >> |
| Miyoshi et al | 2019 | Zambia | 1754 | Retrospective | 18.2 | 42 | 7 | 25 | 43 | 75 | 0 | 75 | Not reported | >> |
| Mary S. et al | 2019 | Colombia | NA | Cross-sectional | NA |  | 1275 | 368 | 1718 | 3901 | 540 | 3361 | Death Certificates | above 22 weeks to 28 days |
| B. Sharma et al | 2020 | India | 5574 | Prospective | 54.4 |  | 217 | 97 | Not included | 314 | 0 | 314 | Obstetrician and doctors managed the woman | >> |
| D. Fabrizio et al | 2020 | Italy | 141,013 | Prospective | 31.9 |  | 402 | 30 | Not included | 443 | 11 | 432 | Neonatologist, Pathologist, and Obstetrician(multidisciplinary audits ) | >> |
| Dase et al | 2020 | Nigeria | 21462 | Retrospective | 55 |  | 365 | 395 | Not included | 1177 | 417 | 760 | Routinely documented | 28 and above weeks of gestation/Birth weight of ≥1000 grams |
| T. Wasim et al | 2020 | Pakistan | 11 850 | Prospective | NA | 58.2 | 167 | 73 | 450 | 690 | 0 | 690 | multidisciplinary group | >> |
| Z. D. Prust et al | 2020 | Suriname | 9089 | Cross-sectional | 14.4 |  | 96 | 11 | Not included | 113 | 6 | 107 | Certificates | above 22 weeks to 28 days |
| Luk et al | 2020 | Hong Kong | 34920 | Retrospective | 2.6 | 3.4 | 91 | 1 | 27 | 119 | 0 | 119 | Team of expert | after 24 completed weeks gestation Neonatal death cases within 28 days of birth |
| Feng et al | 2020 | China | 87,588 | Retrospective | 4.80 |  | 420 | Not included | Not included | 420 | 0 | 420 | Team of expert | After 20 completed weeks of gestation. or birth weight ≥ 350 g if gestational age is unknown. |
| Y.K. Mok et al | 2020 | Hong Kong | 39,625 | Retrospective | 3.7 |  | 132 | 3 | Not included | 145 | 10 | 135 | Clinicians | 24 weeks and above weeks |
| Khulood K. et al | 2020 | Jordan | 10,328 | Prospective | 9.9 |  | 83 | 12 | Not included | 102 | 7 | 95 | obstetrician and Neonatologist | 20 weeks and above /birth weight ≥ 350 g (unknown date) |
| Natasha H. et al | 2021 | Tanzania | 9333 | Prospective | 44 | 71 | 128 | 129 | 202 | 744 | 285 | 459 | Routinely completed by the nurses | 28 weeks and above |
| G.Dagdeviren et al | 2021 | Turkey | 74,102 | Cross-sectional | 6.4 |  | 412 | 46 | Not included | 475 | 17 | 458 | Not reported | 24 weeks and above gestational weeks |
| Shrestha J et al | 2021 | Nepal | NA | Retrospective |  |  | 229 | 59 | 173 | 461 | 0 | 461 | Not reported | 28 weeks and above /birth weight of ≥1000 g |
| WHO | 2021 | North Macedonia | NA | Prospective |  |  | 89 | 6 | 74 | 202 | 33 | 169 | expert team | 22 weeks and above |
| Taweevisit et al | 2022 | Thailand | NA | Retrospective |  |  | 126 | 204 | Not included | 330 | 0 | 330 | Not reported | >> |
| Francesco L. et al | 2022 | Italy | 34,417 | Retrospective | 55 |  | 180 | 11 | Not included | 191 | 0 | 191 | Not reported | >> |
| Salih Metin et al | 2022 | Turkey | NA | Retrospective | NA |  | 119 | 15 | 95 | 229 | 0 | 229 | Not reported | above 24 weeks |
| Manarangi D. et al | 2022 | Solomon Island | 11,056 | Retrospective | 30.8 |  | 166 | 28 | Not included | 341 | 147 | 194 | Not reported | 20 weeks and above/birth weight ≥ 500g |
| Total |  |  | 514989 |  |  |  | 25563 | 6418 | 14835 |  | 1780 | 46816 |  |  |

GA: gestational age, SA: SB: Stillbirth, PMR: perinatal mortality rate, PM: perinatal mortality

Table S3: Maternal condition ICD-PM classification

| ICD-PM maternal condition | Main maternal conditions included in group |
| --- | --- |
| M1: Complications of placenta, cord and membranes | 1. Placenta previa  2. Other forms of placental separation and hemorrhage  3. Placental dysfunction, infarction, insufficiency  4. Fetal–placental transfusion syndromes  5. Prolapsed cord / other compression of umbilical cord  6. Chorioamnionitis  7. Other complications of membranes |
| M2: Maternal complications of pregnancy | 1. Incompetent cervix  2. Preterm rupture of membranes  3. Oligo /polyhydramnios  4. Ectopic pregnancy  5. Multiple pregnancy  6. Maternal death  7. Malpresentation before labor  8. Other complications of pregnancy |
| M3: Other complications of labor and delivery | 1. Breech delivery and extraction  2. Other malpresentation, malposition, and disproportion during labor and delivery  3. Forceps delivery / vacuum Extraction  4. Caesarean delivery  5. Precipitate delivery  6. Preterm labor and delivery  7. Other complications of labor and Delivery |
| M4: Maternal medical and surgical Conditions | 1. Pre-eclampsia / eclampsia  2. Gestational hypertension  3. Other hypertensive disorders  4. Renal and urinary tract diseases  5. Infectious and parasitic disease  6. Circulatory and respiratory disease  7. Nutritional disorders  8. Injury  9. Surgical procedure  10. Other medical procedures  11. Maternal diabetes including Gestational diabetes  12. Maternal anesthesia and analgesia  13. Maternal medication  14. Tobacco / alcohol / drugs of addiction  15. Nutritional chemical substances  16. Environmental chemical substances  17. Unspecified maternal condition |
| M5: No maternal condition | 1. No maternal condition identified or healthy mother |

**Egger test result for stillbirth**

| Regression-based Egger test for small-study effects |
| --- |
| Random-effects model |
| Method: REML |
| Moderators: Stillbirth rate SE Stillbirth rate |
| H0: beta1 = 0; no small-study effects |
| beta1 = 0.00 |
| SE of beta1 = 0.498 |
| z = 0.00 |
| Prob > z = 1.0000 |

**Egger test result for perinatal mortality rate**

| Regression-based Egger test for small-study | effects |
| --- | --- |
| Random-effects model |  |
| Method: REML |  |
| H0: beta1 = 0; no small-study effects |  |
| beta1 = 8.32 |  |
| SE of beta1 = 9.664 |  |
| z = 0.86 |  |
| Prob > z = 0.3891 |  |

Figure S1: Funnel plot of publication bias test among the studies conducted on stillbirth rate

Figure S2: Funnel plot of publication bias test among the studies conducted on perinatal mortality rate

**Sensitivity analysis**

**
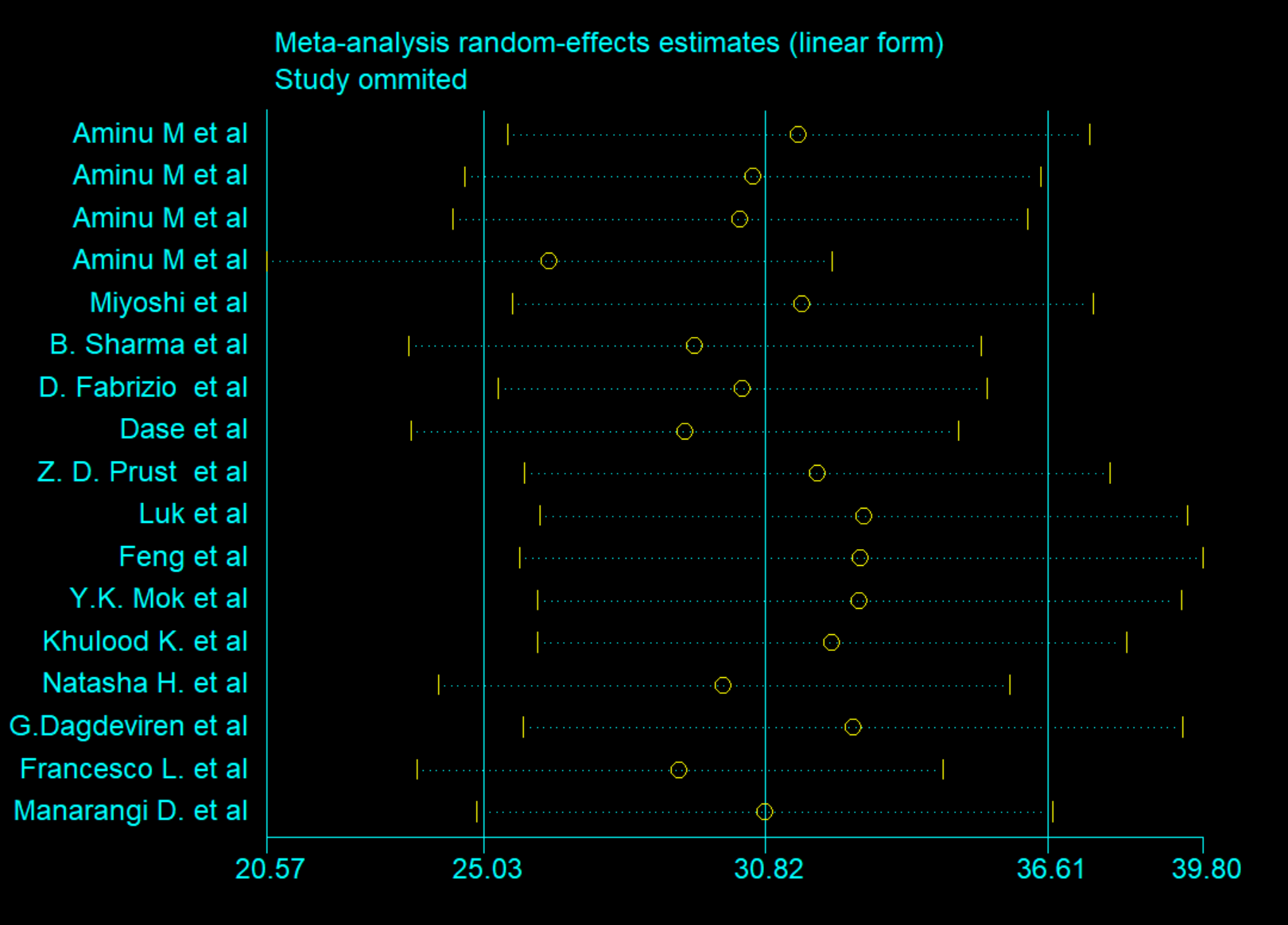
**

Figure S3: Sensitivity analysis result of studies used to estimate the pooled rate of stillbirth

Table S4: Sensitivity analysis result of studies used to estimate the pooled rate of stillbirth

**
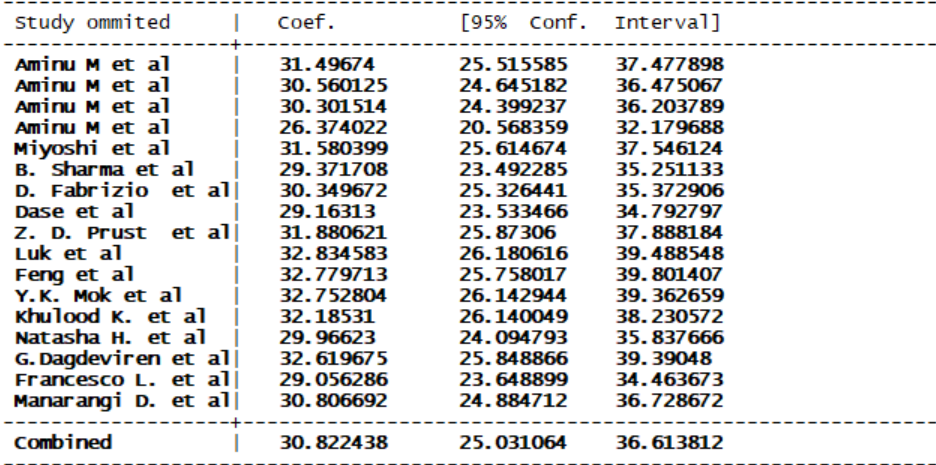
**
